# Orthology-driven mapping of bidirectional promoters in human and mouse genomes

**DOI:** 10.1186/1471-2105-15-S17-S1

**Published:** 2014-12-16

**Authors:** Mary Qu Yang, Laura Elnitski

**Affiliations:** 1 MidSouth Bioinformatics Center, Department of Information Science, George W. Donaghey College of Engineering and Information Technology, University of Arkansas at Little Rock, 2801 S. University Avenue, Little Rock, Arkansas, 72204, USA; 2 Joint Bioinformatics Graduate Program, University of Arkansas at Little Rock and University of Arkansas for Medical Sciences, Little Rock, Arkansas 72204, USA; 3 National Human Genome Research Institute, National Institutes of Health, Rockville, MD 20852, USA

## Abstract

**Background:**

The presence of bidirectional promoters in all vertebrate species suggests that the promoters may be maintained in orthologous positions. Therefore the identification of the comprehensive orthologous mapping of this type promoter across species can facilitate elucidation of regulatory mechanisms controlling bidirectional gene expression. However, the lack of annotation for many transcribed regions in the genome can impact the orthology designation of these promoters. Human and mouse are among genomes that have been relatively well annotated. Thus we used them as models to study the orthologous patterns of bidirectional promoters.

**Results:**

We developed a method to annotate these regulatory regions by confirming the orthology of the genes found on each side of the promoters. In this manuscript we report the cross-species comparisons between human and mouse genomes, where the bidirectional promoter sets regulating UCSC Known Genes and spliced EST annotations were mapped from human to mouse and vice versa. We validate hundreds of orthologous bidirectional promoters through the presence of orthologous flanking gene annotations in the second species. We also show that regulatory activity of these orthologous promoters confers similar gene expression profiles in 21 tissues of human and mouse. In particular, more than one third of human bidirectional promoters annotated from spliced EST annotations regulate ncRNA, of which over 90% are lncRNAs.

**Conclusions:**

Although evolutionary conservation shows a weaker signature in promoters than coding regions, our technique of mapping of orthologous genes shows that most bidirectional promoter arrangements are conserved across human and mouse genomes, suggesting a critical function. In addition, the similar expression patterns of the orthologous gene sets indicate that the regulatory mechanisms remain largely conserved as well.

## Background

Bidirectional promoters are the regulatory regions that fall between pairs of genes, where the 5' ends of the genes within a pair are positioned in close proximity to one another. This spacing facilitates the initiation of transcription of both genes, creating two transcription forks that advance in opposite directions. The formal definition of a bidirectional promoter requires that the transcription initiation sites are separated by no more than 1,000 bp from one another. Using these criteria we have comprehensively annotated the human and mouse genomes for the presence of bidirectional promoters, using *in silico *approaches [1,2]. The identification of these promoters is contingent upon the presence of adjacent, oppositely oriented pairs of genes, whose orthology assignments are quantitatively stronger than noncoding regions. This approach allows us to uniquely identify bidirectional promoters *de novo *[1,3] and does not require tissue-specific epigenetic data that cannot be easily compared across tissues of different species. Genomic annotations used for our identification phase include (1) curated protein-coding gene annotations and (2) spliced ESTs (spESTs) and (3) 5' "end-capped" transcript data, e.g., Cap-Analysis of Gene Expression Database (i.e., CAGE) [4]. The annotations for protein coding genes are robust with certainty and therefore provide a high quality dataset for mapping bidirectional promoters. In contrast, bidirectional promoters supported by RNA evidence alone (as in (2)) have varying levels of evidence, ranging from one characterized transcript to hundreds of them. For this reason, dataset (3) - the CAGE data - provides a stringent level of validation for the start sites of the EST transcripts. As a large class of regulatory sequences, bidirectional promoters exemplify a rich source of unexplored biological information in the human genome. Here, we show that when compared to the mouse genome, these promoters are identifiable as truly orthologous locations, being maintained in regions of conserved synteny (including both genes and the intervening promoter region) that have undergone no rearrangements since the last common ancestor of humans and mice, 75 million years ago. These analyses represent a unique approach to identifying orthologous promoter regions with a high level of certainty.

## Results

### Bidirectional promoter identification

#### Bidirectional gene pairs in human protein-coding genes

**Figure 1:**
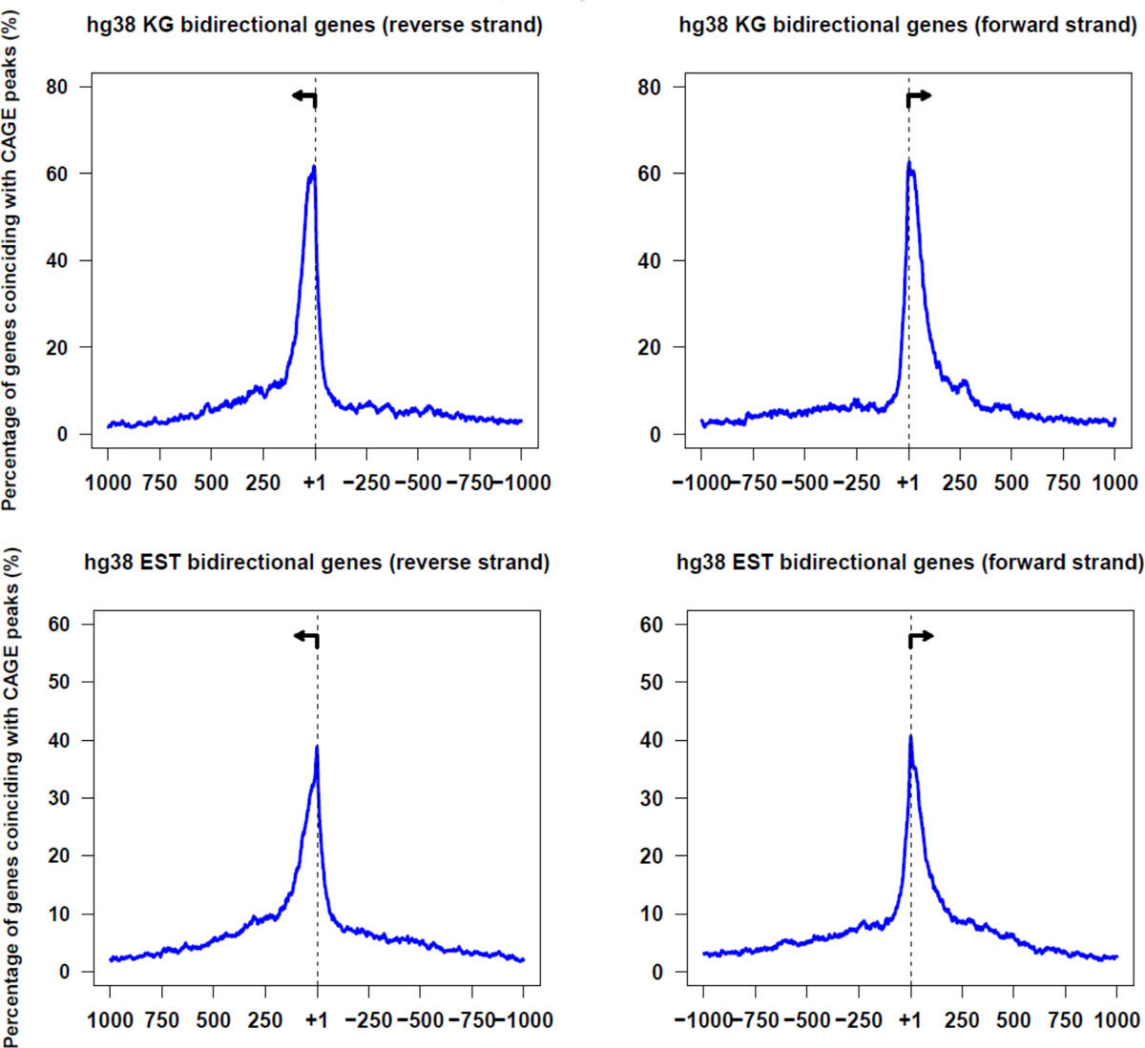
**Validation of 5' gene annotations in human with CAGE tags**. The percentage of the annotated genes coinciding with CAGE peaks in the genomic regions surrounding TSSs. Panel A depicts genes from the UCSC Known Genes annotations. Panel B shows transcripts from the spliced EST dataset. Arrows depict the left and right TSS of bidirectional gene pairs.

We first mapped bidirectional promoters in the human genome. Out of 28,687 protein-coding genes from the UCSC Human Genome Browser (hg38), we found 2,718 bidirectional gene pairs (meeting the 1,000 bp requirement). CpG islands were present at 90.6% of those bidirectional promoters (2,464 / 2,718) compared to 55.3% of non-bidirectional promoter regions (15,943 / 28,687) from the genome. The 5' ends of genes in the bidirectional pairs were validated using RIKEN's CAGE transcripts [5,6]. Cumulatively, the peak position of the CAGE transcript annotations coincided with the annotations of transcription start sites for bidirectional promoters from human Known Genes (Figure 1A).

#### Bidirectional promoters identified by spliced ESTs

A richer source of bidirectional promoter evidence was present in the spliced EST data. This dataset contained more abundant data than the protein-coding gene set, requiring 7 million transcripts to be condensed into unique, non-overlapping loci [1]. The complexity of this data required that we use a stringent approach of classifying potential bidirectional promoters to avoid false positive predictions. We developed and implemented a rigorous mapping procedure to identify such promoters [1]. Using the spEST data from the UCSC Genome Browser (requiring at least one canonical intron) we detected 2,939 additional bidirectional promoters not detectable via the protein-coding gene annotations.

When the transcription start sites of these bidirectional transcripts were compared to the CAGE transcripts, they showed a similar pattern as Known Genes data (Figure 1B). Furthermore, using the CAGE data, we found that 66% of the genes in bidirectional gene pairs exhibited coordinated transcriptional activation, having a CAGE tag at the left and right TSS in the tissues examined.

#### Bidirectional promoter annotation in the mouse genome

**Figure 2:**
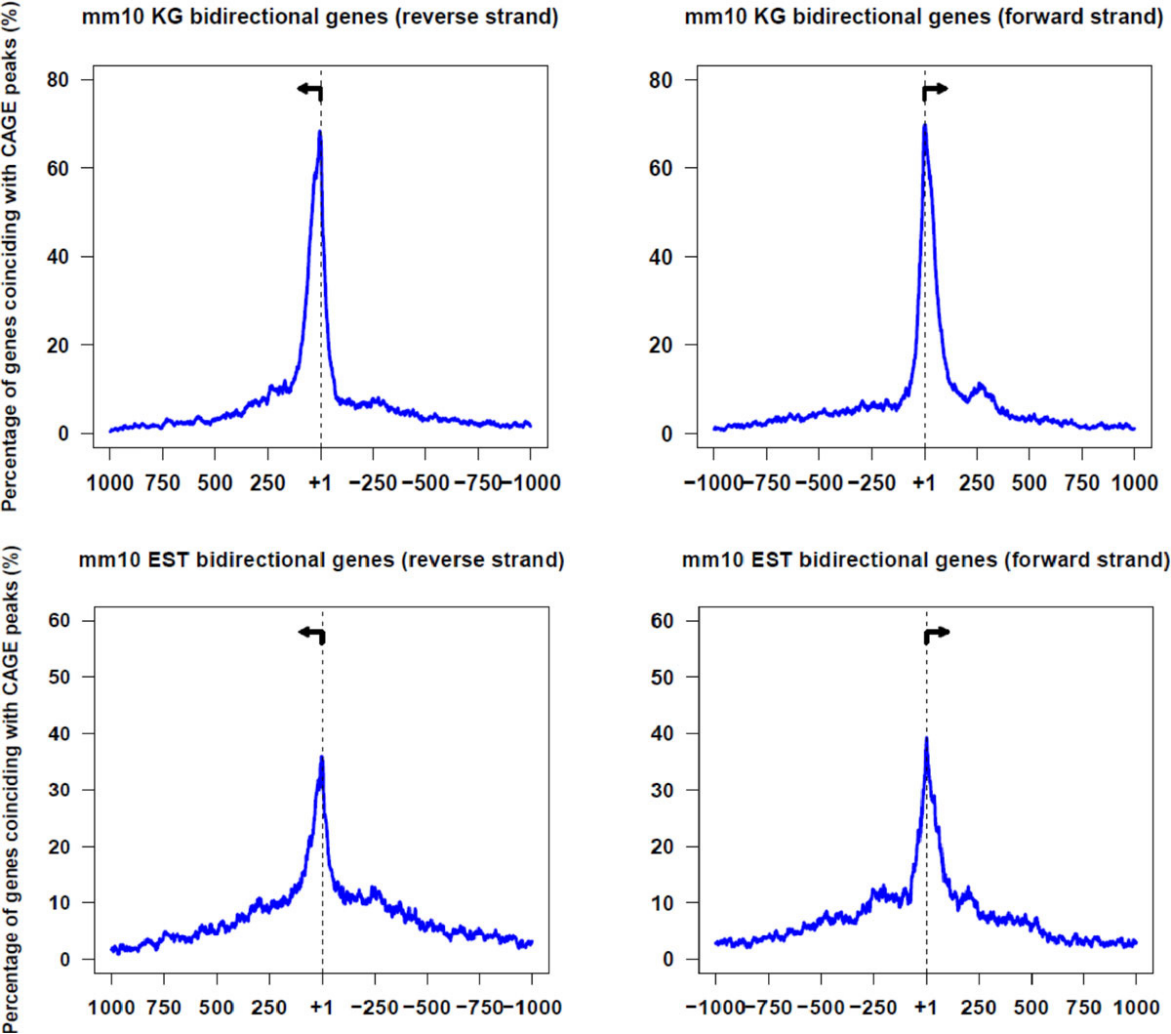
**Validation of 5' gene annotations in mouse with CAGE tags**. The percentage of the annotated genes coinciding with CAGE peaks in the genomic regions surrounding TSSs. Panel A depicts genes from the UCSC Known Genes annotations. Panel B shows transcripts from the spliced EST dataset. Arrows depict the left and right TSS of bidirectional gene pairs.

We obtained 23,618 unique protein-coding gene clusters from the Mouse UCSC Genome Browser (assembly MM10) and identified 1,489 bidirectional gene pairs within the Known Genes annotations. Furthermore, applying our methodology on spESTs (i.e., requiring at least one canonical intron; spEST; also from MM10), we found 732 additional bidirectional promoters were uniquely identified by that dataset. Comparison of the bidirectional gene pairs in Known Genes annotations showed majority of UCSC genes coinciding with CAGE transcript annotations (Figure 2A). The CAGE transcript placement relative to the annotated TSS locations in the mouse spliced EST dataset is shown in Figure 2B. These start sites showed the most variability in the accuracy of the 5' ends of the transcripts of any annotations that we mapped.

### Orthologous bidirectional promoter identification

#### Assigning orthologous regions

As coding regions have the strongest orthologous alignment signal compared with other genomics regions, we used orthology of adjunct gene pairs as anchors to assess the ancestral relatedness of the intervening bidirectional promoters. The orthology of genes was determined using chain and net data from UCSC Genome Browser. Chains in the Genome Browser represent sequences of gapless aligned blocks. Nets provide a hierarchical ordering of those chains. Level 1 chains contain the longest, best-scoring sequence chains that span any selected region. Subsequent levels in the net represent the results of rearrangements, duplications, insertions and deletions that may have disrupted the presence of conserved synteny derived from an ancestral sequence.

#### Confirming orthologous genes

After determining the orthology assignments using the UCSC chains and nets data, we used the Known Gene annotations or spliced ESTs to search the identity of genes within the corresponding region. Known Genes represent protein-coding genes and therefore orthology can be verified by chains and nets alignments, followed by confirmation of protein identity in both species. Spliced ESTs carry less descriptive information than protein coding genes and therefore cross-species comparisons require their presence in an orthologous position, showing conserved synteny of two transcripts forming a divergent pair and meeting the criteria of less than 1,000 bp of intergenic distance between those transcripts. Our method for mapping bidirectional promoters in the spliced EST datasets is described in more detail in a previous publication [1]. When our program verified evidence for orthology and conserved-syntenic gene arrangement, the orthologous bidirectional promoter was confirmed. After orthologous assignments were confirmed in mouse for pairs of human genes, the reciprocal assignments were analyzed from mouse back to human.

#### Orthology mapping of bidirectional promoters from human to mouse

Within a species, annotated transcripts provide critical evidence for identifying bidirectional promoters. Across species, over 90% of the human and mouse genomes can be partitioned into corresponding regions of conserved synteny [7]. We hypothesized that conserved synteny of bidirectional gene pairs predicts the presence of orthologous bidirectional promoters. Thus missing annotations at the 5' ends of genes could be predicted from comparisons to a second species. We developed a methodology to examine orthologous locations of the pairs of genes and their intergenic promoter regions in a second species as a method of prediction, discovery and validation.

As mentioned, the bidirectional promoter sets were partitioned into those regulating protein-coding genes and those identified from the spliced EST evidence. For example, the protein coding set was defined by annotations in the Human UCSC Genome Browser annotations for Known Genes. This highly curated dataset provided a robust test of the hypothesis for cross-species mapping (see Methods). The 2,718 bidirectional promoters from human were classified into five categories that describe the state of the promoter in the mouse genome. These included:

1. the human gene has an ortholog in mouse and that ortholog has a bidirectional partner within 1,000 bp that is an ortholog of the gene partner in human

2. the human gene has an ortholog in mouse and that ortholog has a bidirectional partner within 1,000 bp that is not an ortholog of the gene partner in human

3. the human gene has an ortholog in mouse and that ortholog is missing a bidirectional partner within 1,000 bp

4. a non-orthologous gene was mapped to the corresponding mouse location

5. no orthology was recorded in the mouse genome

**Figure 3:**
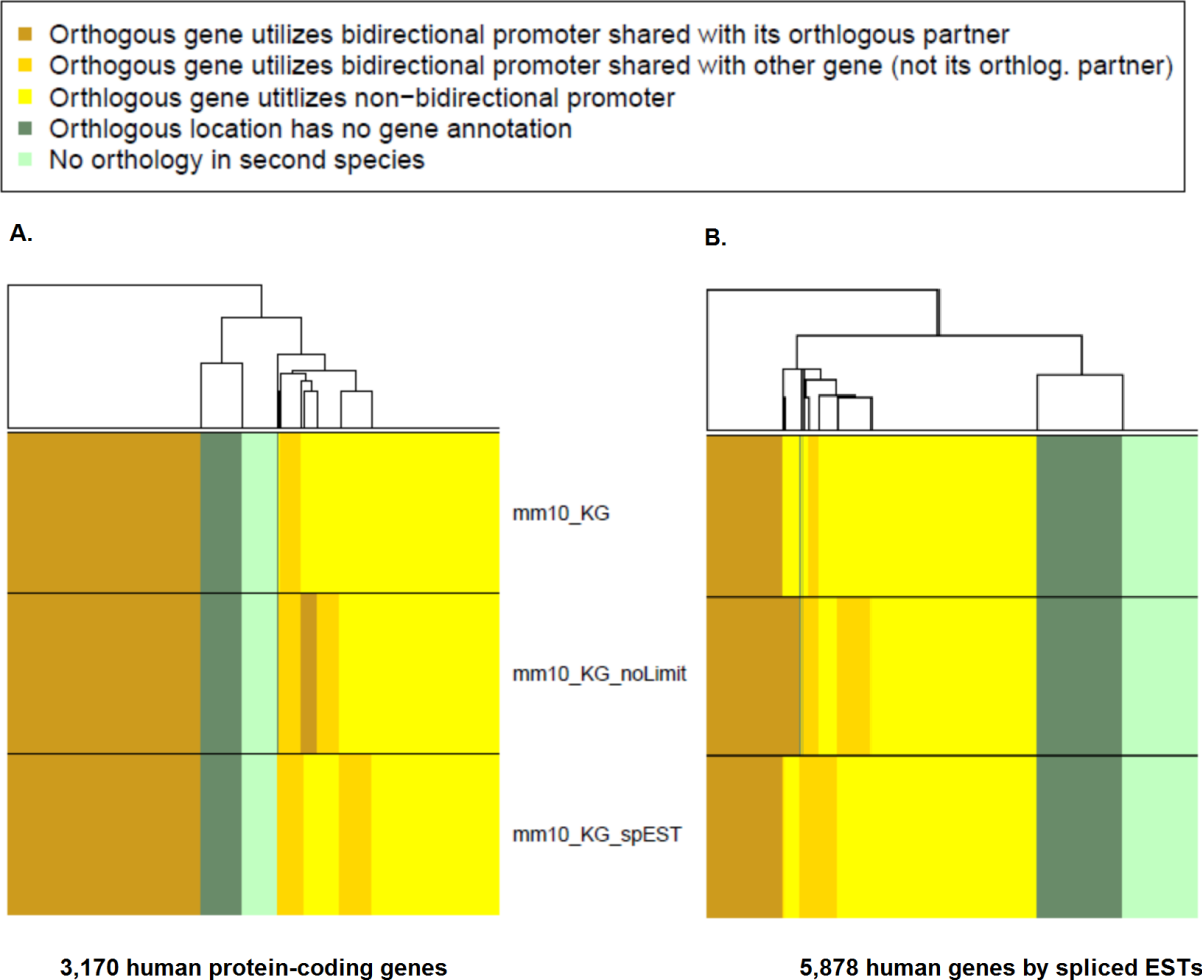
**Cross-species mapping of human bidirectional promoters**. (A) The 1,585 protein-coding gene pairs are represented as 3,170 individual genes for clarity of the analysis. These genes were mapped to the mouse genome to identify the presence of orthologous bidirectional promoters. If the mouse annotations independently supported a bidirectional promoter at the orthologous location, and the genes on each side of the mouse promoter were shown to be orthologs of the human gene pair, then promoter orthology was confirmed. The annotations in the mouse included the mouse Known Gene (mm10_KG) where no more than 1,000 bp separated the transcription start sites in mouse as well as in human. Results using an unlimited distance between the genes in mouse are shown in the panel mm10_KG_NoLimit. A composite dataset of the mouse Known Genes and mouse ESTs occupies the lower panel, MM10_KG_spEST. (B) The 2,939 gene pairs represented by 5,878 individual genes in the human spliced ESTs were mapped to the mouse genome to identify the orthologous bidirectional promoters. The three panels use the same validation approach as in panel A, shown by the same labels.

Figure 3A shows the results of mapping the human set of bidirectional promoters to the mouse genome using the Known Genes dataset. Over 39.4% of the bidirectional promoters were validated as orthologous in mouse (gold bars). The first panel shows predictions that were validated in mouse by the mouse Known Genes annotations (MM10_KG). The second panel shows that additional orthologous promoter regions, up to 42.8%, could be identified when the strict 1,000 bp limit was rescinded for the mouse annotations (MM10_KG_NoLimit, Additional file 1). This result confirmed that the orthologous protein-coding genes were present and suggested that their 5' UTRs were incompletely annotated, creating an intergenic distance larger than allowed by the 1,000 bp criterion. Confirmation of this explanation will require further experimental analyses such as 5' RACE. The third panel combines all data, showing that additional bidirectional promoters were also identified through combining the Known Gene and EST annotations.

Figure 3B shows the results of mapping bidirectional promoters from the human EST dataset to the mouse. In this case, fewer orthologous promoters were identified, 12.4%; nevertheless, the same trends were observed as before. For example, allowing a distance larger than the 1,000 bp intergenic space between the transcription start sites in the mouse genes validated a larger number of orthologous gene positions, 15.6%. A large number of examples had evidence for only one orthologous transcript, 23%. The combination of the two datasets (Known Genes and spliced ESTs) increased the number of orthologous promoters modestly.

#### Orthology mapping of bidirectional promoters from mouse to human

**Figure 4:**
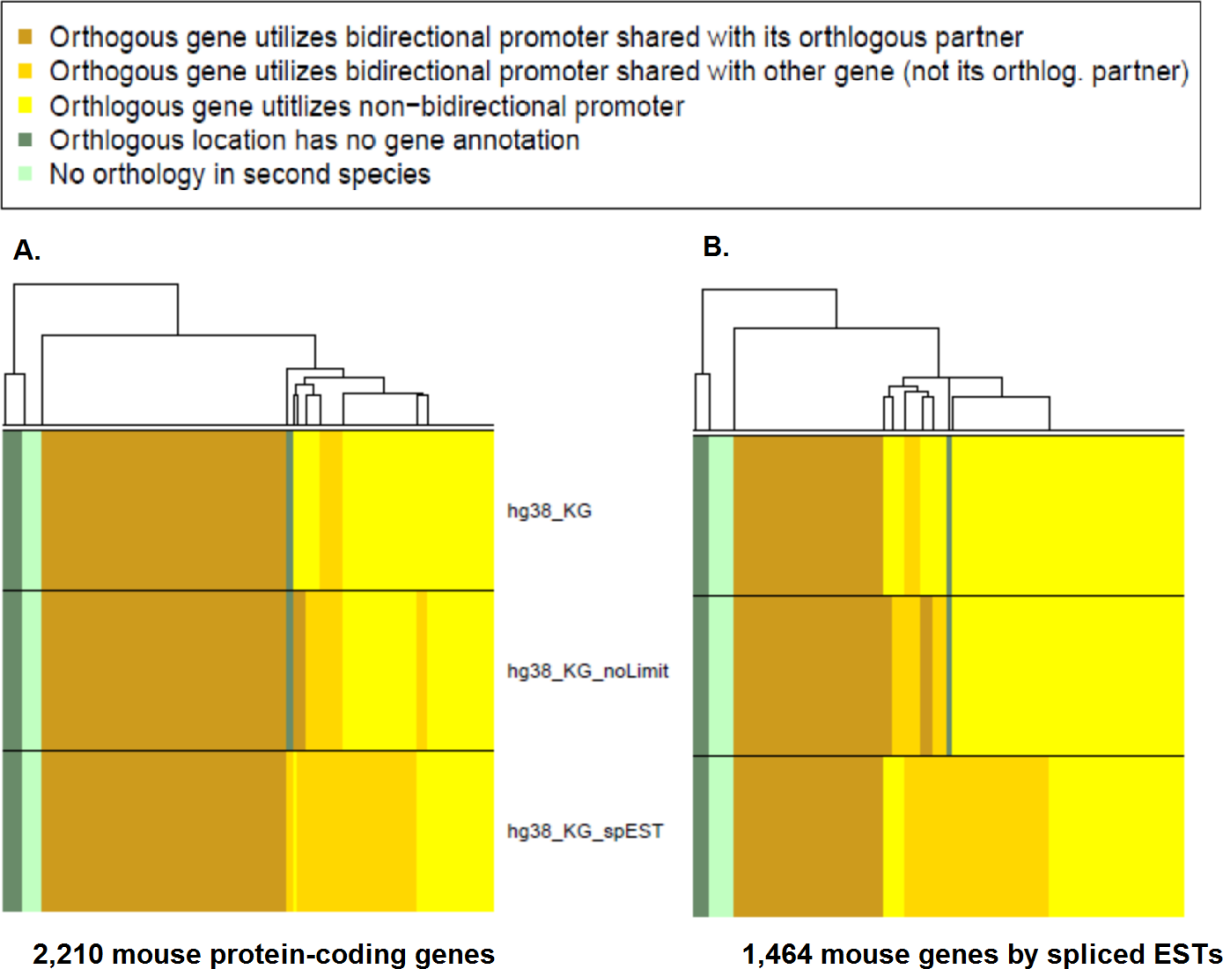
**Cross-species mapping of mouse bidirectional promoters**. (A) The 1,105 protein-coding gene pairs are represented as 2,210 individual genes. These genes were mapped to the human genome to identify the presence of orthologous bidirectional promoters. If the human annotations independently supported a bidirectional promoter at the orthologous location, and the genes on each side of the human promoter were shown to be orthologs of the mouse gene pair, then promoter orthology was confirmed. The annotations in the human included the human Known Genes (hg38_KG) where no more than 1,000 bp separated the transcription start sites in human as well as in mouse. Results using an unlimited distance between the genes in human are shown in the panel hg38_KG_NoLimit. A composite dataset of the human Known Genes and human ESTs occupies the lower panel, hg38_KG_spEST. (B) The 732 gene pairs represented by 1,464 individual genes in the mouse spliced ESTs were mapped to the mouse genome to identify the orthologous bidirectional promoters. The three panels use the same validation approach as in panel A, shown by the same labels.

The same procedure was repeated by comparing the mouse bidirectional promoters to the annotated human genome. Figure 4A shows the results of mapping the Known Genes promoter set from mouse to human, where over 50% of the promoters were validated by existing annotations in the human genome. These annotations were from the human Known Genes (hg38_KG) containing a 1,000 bp intergenic limit between them, or having no limit on the intergenic distance, denoted as hg38_KG_NoLimit. Removing the limits identified 52.5% of the promoters as orthologous. Figure 4B shows the procedure performed using the bidirectional promoter set detected from the mouse EST annotations. As also seen with Figure 3B, many fewer promoters from the EST predictions were validated in the second species (30.7%). The number increased to 35% when the 1000 bp limited was removed (Figure S1, Additional file 1). These data show that promoter annotations identified in mouse ESTs can robustly validate some human promoters, and that comprehensive mapping of these promoters is aided by multiple lines of transcript evidence.

#### Distribution of orthologous bidirectional promoters on chromosomes

**Figure 5:**
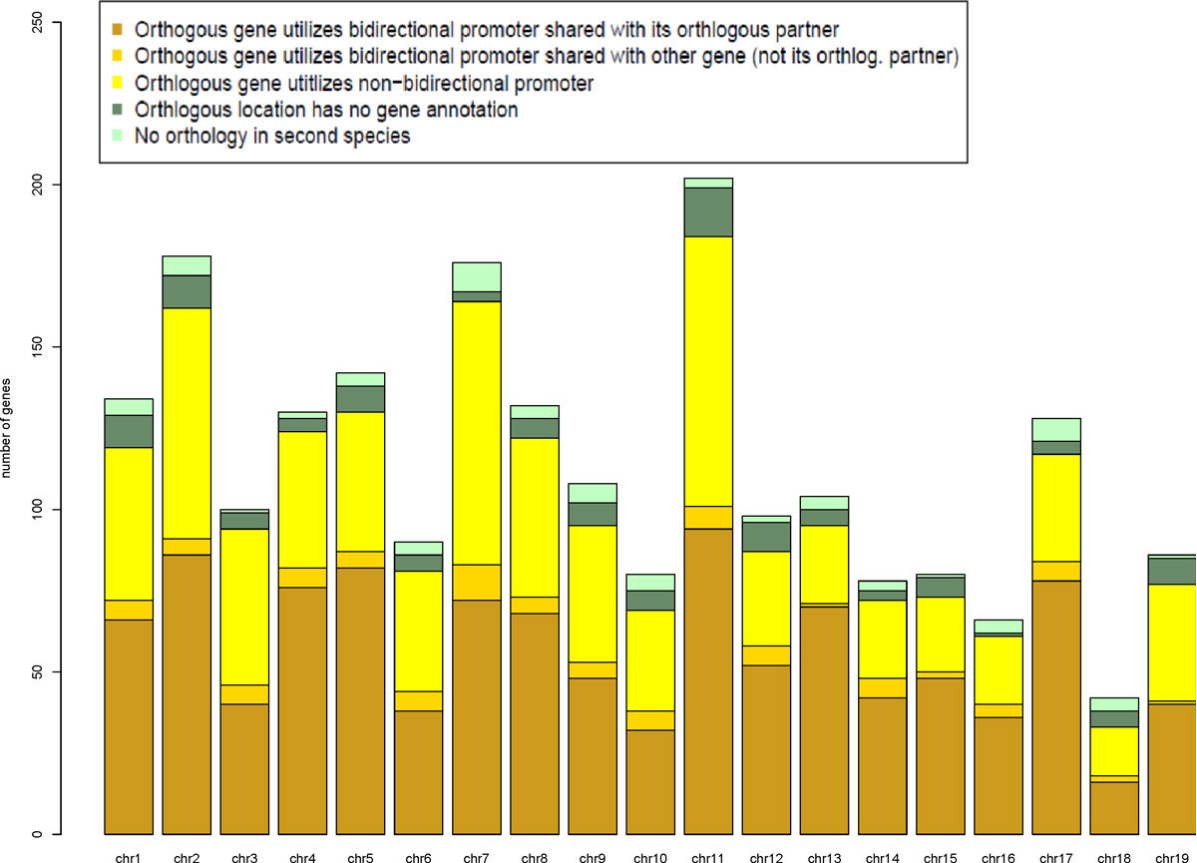
**Outcome of mapping orthologous bidirectional promoters from human to mouse**. The Known Genes dataset in humans was mapped to orthologous positions in mouse and validated using Known Genes annotations in mouse. Arranged by chromosome, bidirectional promoters were validated as orthologous when orthologous genes were present on both sides of the promoter in the second species. These examples are colored brown. Instances with one orthologous gene flanking the promoter region in the mouse genome are colored yellow. Green panels lack evidence from either gene of the pair, within the 1000 bp intergenic region that is orthologous in the mouse.

On a per chromosome basis, genes regulated by bidirectional promoters were not evenly distributed in either the human or mouse genomes (Figure 5, Additional file 2). However, their appearance was consistent with the allocation of genes per chromosome. For example, in the human genome, chromosome 13 has the lowest gene density (6.5 genes per Mb) among sequenced human autosomes [8], as well as one of the lowest numbers of bidirectional promoters. In contrast, chromosome 19 is the most gene-rich of all human chromosomes [8] and has a high number of bidirectional promoters. Chromosome 16 had the highest ranking, containing over 52% of bidirectional promoters in human that were confirmed as orthologous in mouse. Those promoters currently showing no orthologous evidence represent either species-specific differences between human and mouse gene sets (true negatives) or missing annotations from the Known Genes dataset in mouse (false negatives). We observed a higher overall confirmation of orthologous bidirectional promoters when mapped from mouse to human (Additional file 3), suggesting that the annotations may be more complete in human, and the mapping from human to mouse failed more often due to missing gene annotations in mouse. We will continue to update the datasets as gene annotations continue to be refined.

### Orthologous promoters exhibit functional correlations

**Figure 6:**
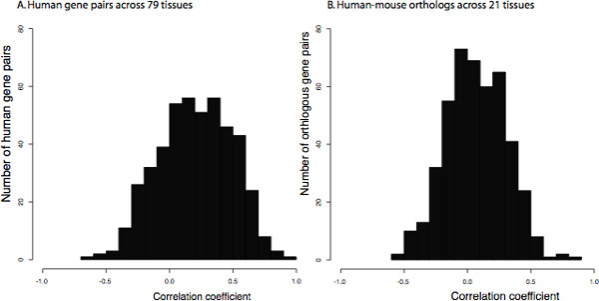
**Expression correlation of genes regulated by orthologous promoters**. Figure 6A shows the expression correlation of bidirectional gene pairs in 79 human tissue types. Figure 6B shows expression correlation of human gene pairs in 21 mouse tissues, regulated by orthologous bidirectional promoters.

The presence of orthologous regulatory regions provided an opportunity to dissect the similarity in gene expression conferred across species by bidirectional promoters. By scanning the Novartis Expression Atlas 2 dataset [9] for human and mouse orthologs in our set (and using the median expression ratio), 456 genes and their confirmed orthologs were compared. Figure 6A shows that human bidirectional gene pairs are more likely to be co-expressed when examined in 79 different tissue types. A total of 21 tissues of the same identity in human and mouse were also examined. Figure 6B shows a shift toward correlated expression among orthologs. However, some negative correlations were also detected.

## Discussion

We have utilized the unique properties of bidirectional promoters to map orthologous regulatory regions. These promoters are flanked on each side by a spliced transcript. Therefore the presence of the orthologous genes in the same arrangement in another species identifies the intergenic promoter region as the orthologous promoter region. We have used this approach to map promoters from human to mouse without the aid of regulatory region sequence conservation to identify the orthologous promoter elements. Nevertheless sequence alignments were very important in defining the regions of orthology and conserved synteny. We show that orthologous regulatory regions can be identified using annotations of UCSC Known Genes or spliced ESTs. Furthermore, the combination of these datasets reveals additional promoter regions. By validating the predictions in the second species, we confirmed that bidirectional promoters are present in orthologous positions in mammalian genomes. Furthermore, we postulate that regions containing one of the genes, but not both, are likely to be missing the annotations for the partner gene. Thus we anticipate that as annotations grow more populated and refined, the data shown in our heat maps will confirm orthology at even more promoters.

## Conclusions

Bidirectional promoters are enriched in mammalian genomes. Our approach of investigating the orthology of bidirectional promoters reveals thousands of examples of this type of regulatory structure maintained through evolutionary selection. By combining spliced ESTs and Known Genese, we identified a larger and more comprehensive set of bidirectional promoters. We subsequently found that many of these spliced ESTs represent non-coding RNAs (ncRNA). This is consistent with recent reports that the majority of long non-coding RNA (lncRNA) are flanked by bidirectional promoters [10-12]. Thus, understanding regulatory mechanisms of bidirectional promoters can be useful in investigating ncRNAs whose functions remain largely unknown. The different types of bidirectional promoters we record based on annotation and orthology allow us to address the diversity of biological functions of these promoters.

## Methods

### Bidirectional gene pairs in human and mouse protein-coding genes

We downloaded protein coding gene and spliced EST annotations from UCSC Genome Browser [13]. Assembly hg38 and mm10 were used for human and mouse genomes, respectively. We used 1,000 bp as the intergenic distance cut-off in defining a bidirectional promoter between two adjacent gene pairs. The major steps of identifying bidirectional promoters from spEST include extracting all bidirectional promoters genome wide, collapsing overlapping candidate promoter regions, filtering out false positives from the dataset and assigning confidence levels to these promoters.

The CAGE peaks was obtained from FANTOM5 (Functional Annotation of the Mammalian Genome) datasets [5,6]. We converted the coordinates of CAGE peaks from hg19 to hg38 using liftOver [14].

### Orthology mapping of bidirectional promoters

A multi-stage approach to mapping orthology at bidirectional promoters was developed. Orthology assignments are strongest in coding regions. Therefore we began by mapping single human genes regulated by bidirectional promoters onto the mouse genome. Orthology assignments were determined using the "chains and nets" data from the UCSC Human Genome Browser MySQL tables [15]. We used orthologous regions present in only level 1 chains and excluded any other levels, which contained both paralogous (duplicated during evolution) and orthologous sequences. Level 1 alignments also contained extremely long stretches of genes in conserved synteny (i.e. same gene identity and location) between species. Given a human gene, our approach examined whether it fell within an orthologous region defined by level 1 alignment data without knowledge of the exact position within an alignment or relative to a gap. In a subsequent step, we intersected the positions of gaps and exons of each gene to ensure that the exons fell into alignable positions across species.

## Competing interests

The authors declare that they have no competing interests.

## Authors' contributions

LE and MQY conceived and designed the project. MQY conducted experiments and implemented the project. MQY and LE performed analyses. LE and MQY wrote the manuscript and both authors approved the final manuscript.

## Supplementary Material

Additional file 1 Figure S1: Mapping orthologous bidirectional promoters across species. Click here for file

Additional file 2 Outcome of mapping orthologous bidirectional promoters from mouse to human. Click here for file

Additional file 3 **Number of orthologous bidirectional promoters in different chromosomes**. Click here for file
