# Supplementary figures and images for: Orthology-driven mapping of bidirectional promoters in human and mouse genomes
Source: BMC Bioinformatics. 2014 Dec 16;15(Suppl 17):S1. doi: 10.1186/1471-2105-15-S17-S1 (PMC4304189; doi:10.1186/1471-2105-15-S17-S1)

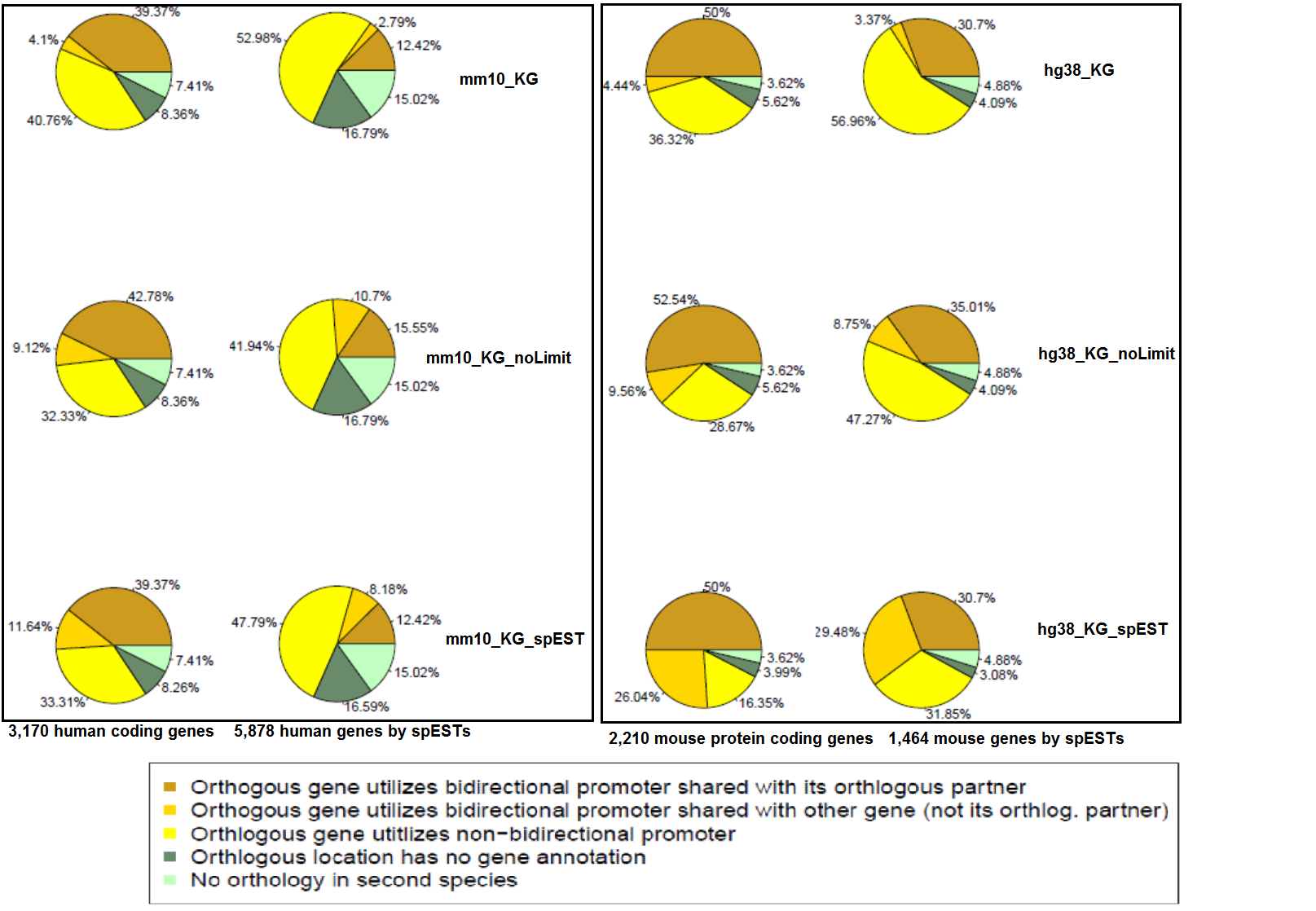

Supplement: Additional file 1 — Figure S1: Mapping orthologous bidirectional promoters across species. [file 1471-2105-15-S17-S1-S1.png]

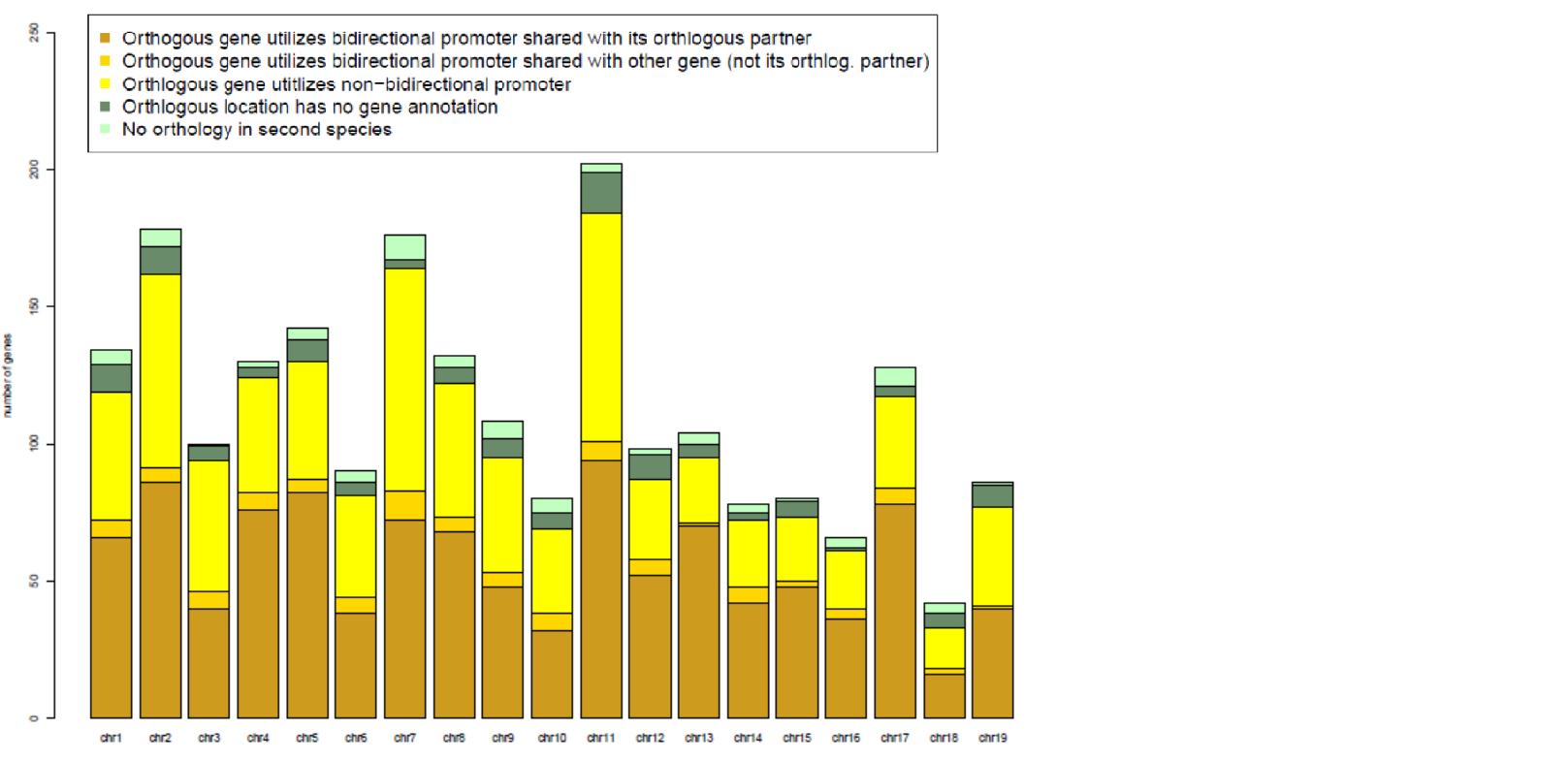

Supplement: Additional file 2 — Outcome of mapping orthologous bidirectional promoters from mouse to human. [file 1471-2105-15-S17-S1-S2.png]
