# Supplementary material for: Orthology-driven mapping of bidirectional promoters in human and mouse genomes
Source: BMC Bioinformatics. 2014 Dec 16;15(Suppl 17):S1. doi: 10.1186/1471-2105-15-S17-S1 (PMC4304189; doi:10.1186/1471-2105-15-S17-S1)

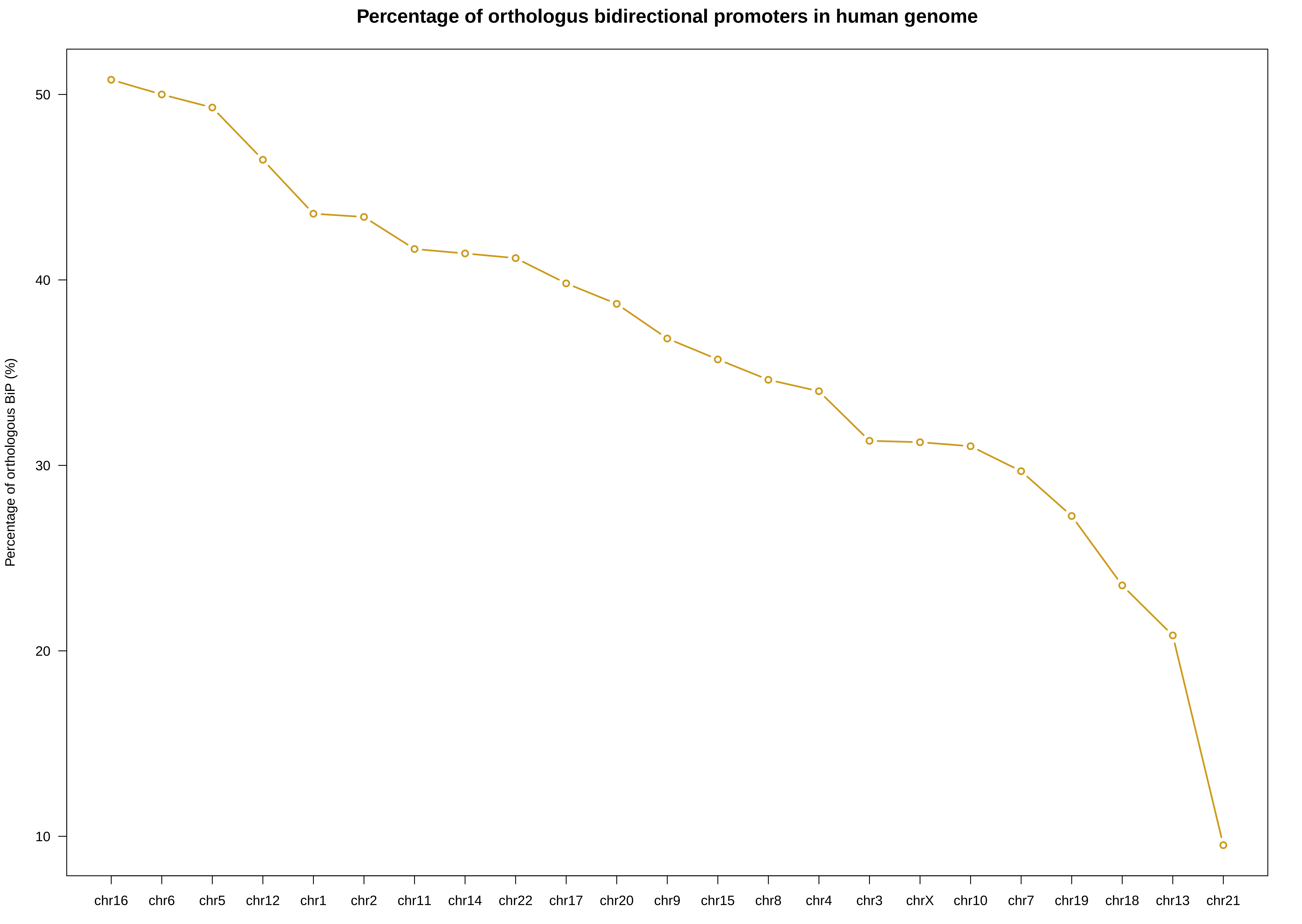
Percentage of orthologus bidirectional promoters in human genome
Percentage of orthologous BiP (%)
50
40
30
20
10
chr16
chr6
chr5
chr12
chr1
chr2
chr11
chr14
chr22
chr17
chr20
chr9
chr15
chr8
chr4
chr3
chrX
chr10
chr7
chr19
chr18
chr13
chr21

Percentage of orthologus bidirectional promoters in human genome

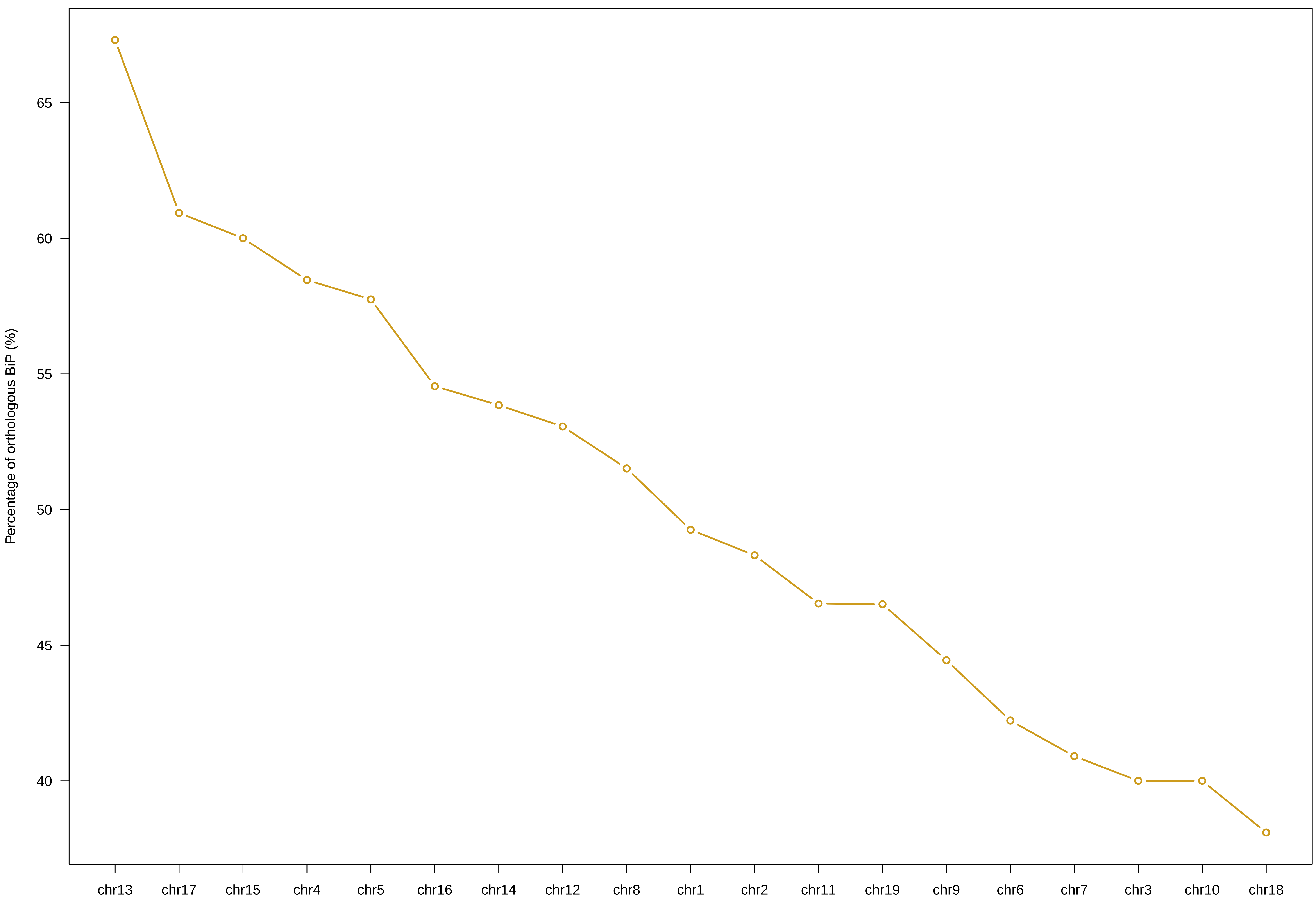

Supplement: Additional file 3 — Number of orthologous bidirectional promoters in different chromosomes. [file 1471-2105-15-S17-S1-S3.pdf]
